# Authenticity in Objects and Activities: Determinants of Satisfaction with Virtual Reality Experiences of Heritage and Non-Heritage Tourism Sites

**DOI:** 10.1007/s10796-022-10286-1

**Published:** 2022-05-23

**Authors:** Kichan Nam, Christopher S. Dutt, Jeff Baker

**Affiliations:** 1 School of Business Administration, American University of Sharjah, Sharjah, United Arab Emirates; 2 Al Rayyan International University College, Doha, Qatar & Emirates Academy of Hospitality Management, Dubai, United Arab Emirates

**Keywords:** Virtual reality (VR), Tourism, Heritage, Non-heritage, System quality, Authenticity, Presence, Satisfaction

## Abstract

Virtual Reality (VR) is becoming an increasingly important technology in a host of industries, including tourism. VR can provide virtual experiences before, during, or in lieu of real-world visits to tourism sites. Hence, providing authentic experiences is essential to satisfy guests with the site and technology. This study analyzes survey data using PLS to identify the determinants of satisfaction with non-immersive VR experiences of heritage and non-heritage tourism sites. Results from 193 subjects reveal the linkages between system quality, object-related authenticity, activity-related authenticity, and presence, as well their relationship with satisfaction.

## Introduction

Virtual Reality (VR), the use of computer technology to create a simulated visual environment (Bardi, 2019; Beck et al., 2019), is becoming an increasingly important and widely used technology (Bednar & Welch, 2020). Valuable applications of VR are being found across multiple industries and disciplines (Hyun & O’Keefe, 2012). VR is being used for data visualization – not only for traditional quantitative data, but also for architectural and engineering data. In medicine, VR is being used to train physicians for surgery. In pharmaceutical research, it is being used to visualize the chemical compounds being created. In psychology, VR is providing insight into human interactions as well as helping patients conquer certain phobias (Diemer et al., 2015). VR is being used in aviation, in the military, police forces, and in broader educational fields (Akhtar et al., 2021; Paszkiewicz et al., 2021) as an alternative to training in live situations where costs are high and dangers may be present.

VR is also being used in tourism, where restauranteurs, hoteliers, and managers of tourist sites can provide potential guests with an opportunity to preview their experience before visiting (Guttentag, 2010) or enhance their experience at tourist sites (S. J. Lee, 2017). VR may sometimes act as a substitute for real-world tourism, but increasingly VR has its own purpose and its own distinct benefits (Cheong, 1995; Mura et al., 2017). VR is an effective planning tool allowing tourists to preview destinations before travelling (Akhtar et al., 2021; Beck et al., 2019; Guttentag, 2010). Post-travel, VR can enable tourists to recall memories and pleasant experiences, which may lead them to revisit the location (Tussyadiah et al., 2018). Additionally, it is possible to experience places where accessibility is limited (Guttentag, 2010). At the time of writing, this includes accessing locations where restrictions have been imposed to curb the spread of COVID-19 (Akhtar et al., 2021; Gittens, 2021; Radermecker, 2021). Furthermore, some people simply enjoy VR content itself without any specific plans to travel in the future. Thus, in tourism VR is seen as a supplementary tool that enhances or reinforces a real experience. It allows tourists to experience objects and places through virtual spaces.

The increasing use of VR has called into question how users perceive and assess the genuineness of what they are viewing, and what impact this has on their experience and satisfaction. While authenticity research has been conducted to explore these relationships at real-world sites, virtual sites remain under-investigated.

Authenticity is a relatively complex construct, one that is used to refer to how genuine visitors believe an item or experience is (Skinner et al., 2020). It is necessary to understand how users view the authenticity of objects and experiences because of the positive impact authenticity has on satisfaction (Cho, 2012; Moscardo & Pearce, 1986; Park et al., 2019; Sylaiou et al., 2010; Wu et al., 2019; T. Zhang et al., 2018), memory and learning (Moscardo & Pearce, 1986), perceived value (S. Lee et al., 2016), perceived quality (Domínguez-Quintero et al., 2020), and revisit intentions (Park et al., 2019). Analysis of authenticity has – particularly in tourism research – frequently been focused on heritage sites (Guttentag, 2010; Schwan & Dutz, 2020; Wu et al., 2019; T. Zhang et al., 2018).

Heritage tourism, which covers visits to places of cultural and historical significance, is sensitive to visitor’s perceptions of authenticity, making authenticity an important component of visitation to heritage sites (Schwan & Dutz, 2020; Wu et al., 2019; T. Zhang et al., 2018). Researchers continue to call for further exploration into tourists’ perceptions of and relationship with the VR sites they visit, including how authentic they perceive the VR sites (Hunter, 2021; Nuryanti, 1996; Poria et al., 2004).

Extensive research on authenticity at heritage sites does not extend to non-heritage sites, however (Duan et al., 2019; Milman, 2013; Waysdorf & Reijnders, 2018). This is a noteworthy omission since the identified benefits of authentic displays – including greater satisfaction, perceptions of quality, and repeat visitation – are desirable for both heritage and non-heritage sites (Duan et al., 2019). Therefore, authenticity of VR is important at both heritage and non-heritage VR sites, and the understanding of authenticity would be improved by comparing perceptions of authenticity at both. Given the prevalence of discussion and debate around the authenticity of cultural and historical assets and sites, it is not surprising that the debate should continue as virtual heritage and non-heritage sites have become accessible online using VR technology (Mura et al., 2017).

The objective of this study is to investigate the relationship between authenticity and satisfaction in two different contexts: heritage VR sites and non-heritage VR sites. Addressing this research objective can provide significant theoretical and practical insights. Theoretically, understanding how VR affects perceptions of authenticity can enhance the understanding of antecedents of authenticity and impacts of VR technology at a range of tourism sites. The impact of VR on perceptions of authenticity can yield insight into understanding how a high degree of authenticity is achieved. Practically, results can provide insight to support the appropriate use of VR at tourism sites. This can benefit management of heritage and non-heritage sites by providing recommendations of best cases when to implement VR and how to promote greater satisfaction and repeated site visits.

The paper proceeds as follows. In Section 2, we review literature on VR, defining VR itself, and observing the importance of presence and authenticity in VR experiences. In Section 3, we present our research model, one that investigates the determinants of satisfaction with VR experiences of heritage and non-heritage tourism sites. Constructs are presented and hypotheses are formally developed. In Section 4, we describe our sample, data collection procedures, and instrument development. Questionnaires were administered to 193 subjects after their virtual visits to three heritage or three non-heritage sites. Section 5 describes our PLS analysis and results, including differential findings at heritage and non-heritage tourism sites. Section 6 interprets our findings and discusses the theoretical and practical implications of our study. Our primary contribution extends the understanding that authenticity influences satisfaction, providing specific subtypes of authenticity that influence satisfaction with VR experiences at heritage and non-heritage tourism sites. We also mention limitations of the current study and opportunities for future research before concluding.

## Literature Review

### Virtual Reality

Virtual reality (VR) is defined in several ways. At its most basic, VR describes computer systems that provide simulated experiences (Beck et al., 2019). More specifically, VR is a “computer-generated three-dimensional environment that one can navigate and possibly interact with, resulting in real-time stimulation of one or more of the user’s five senses” (Guttentag, 2010). Users should feel transported from a physical world to a simulated one by the computer-generated medium (Hobson & Williams, 1995). The simulated environment can lead users to sense that they are present in another location, perhaps in an invented digital location, or perhaps even somewhere else in the real world (Desai et al., 2014).

VR experiences can be classified into three subtypes (Beck et al., 2019). Non-immersive VR (niVR) refers to a virtual technology using conventional computer screens or smartphones that enable the users to navigate locations with 360-degree views, often using a mouse, keyboard, touchpad, or touchscreen. Semi-immersive VR (siVR) is for multi-user environments where images are projected to large screen monitors or walls around the users. Fully-immersive VR (fiVR) uses head-mounted displays (HMDs) where users can be completely isolated from the real world by watching computer-generated views through the device (Beck et al., 2019).

VR research began in earnest in the early 2010s. An early overview explored the potential impact of VR on tourism, identifying six areas where its value should be investigated: planning and management, marketing, entertainment, education, accessibility, and heritage preservation (Guttentag, 2010). Additionally, this study raised the issue of authenticity as an important one for examination. A user’s perception of authenticity can differ based on the originality of the VR object, as well as the user’s personal characteristics, past travel experience, and tourism style. Perception of authenticity can be influenced even by different cultures or locations as well.

A subsequent empirical study examined the impact of VR telepresence on virtual cognitive image and virtual affective image, both of which affect virtual conation (Hyun & O’Keefe, 2012). This model also tested whether telepresence is affected by offline travel information and web-mediated virtual information. Results indicated that web-mediated virtual information positively affects telepresence, and that telepresence positively influences virtual cognitive image and virtual conation.

Other researchers developed a framework to identify factors that affect travelers’ experience and intention to visit in a 3D virtual world (Huang et al., 2005). The technology acceptance model and hedonic theory were employed. Enjoyment, positive emotions, emotional involvement, and flow were hypothesized to influence behavioral intention to visit. Results indicated that perceived usefulness is positively associated with behavioral intention, but perceived ease of use is not associated with behavioral intention in a virtual world. Also, it was found that positive emotions, emotional involvement, and flow have positive relationships with behavioral intention.

Another group of researchers investigated the relationship among VR presence, VR enjoyment, attitude change, and visit intention (Tussyadiah et al., 2018). Results showed that VR presence positively affects VR enjoyment and attitude change while VR enjoyment also positively affects attitude change.

In VR-related tourism research, the concept of presence frequently receives attention. Presence explains the feeling of “being there” in a specific location, whether the location is a computer-visualized real-world location or a wholly synthetic computer-generated world (Ijsselsteijn & Riva, 2003). More precisely, presence is “the sense of being in a virtual experience rather than in the place in which the participant’s body is actually located.” (Sanchez-Vives & Slater, 2005). Presence is positively influenced by web-mediated virtual information, but not by offline information (Hyun & O’Keefe, 2012). Presence itself influences the enjoyment of the VR experience, attitude toward the tourism site, and the intention to visit the site (Jung et al., 2016; Tussyadiah et al., 2016, 2018). Here again, researchers have called for additional focus on the outcomes of a high degree of VR presence, and observed that presence may offer benefits to tourism practitioners as they promote their services, products, and tourism sites (Beck et al., 2019).

Common themes of VR studies are apparent in retrospect (Beck et al., 2019). The most common dependent variables are satisfaction with VR and intention to visit. With respect to these two dependent variables, various independent variables such as presence, enjoyment, involvement, and immersion, were included in the research models. Prior studies have focused on the adoption of VR and have proven that VR has a potential positive impact on tourists and consumers.

### Authenticity

Authenticity is an important topic in tourism research because it is theoretically understood to influence tourists’ satisfaction with an attraction, site, or experience (Cho, 2012; Moscardo & Pearce, 1986; Park et al., 2019; Sylaiou et al., 2010; Wu et al., 2019; T. Zhang et al., 2018). The study of authenticity emerged from research on visitors’ perceptions of museums (Trilling, 1972), and has since been extended to tourism objects and experiences in general (Wang, 1999). Tourists’ perceptions of art, artifacts, food, clothing, ceremonies, festivals, and attractions are described in terms of their genuineness, accuracy, and realness, and thus on a continuum from authenticity to inauthenticity (Sharpley, 2018).

Authenticity is both object-related and activity-related. Object-related authenticity, as the name indicates, refers to the authenticity of objects such as art, artifacts, or buildings. It has an objective, factual aspect as well as a subjective, constructive aspect. For instance, Da Vinci’s Mona Lisa in the Louvre in Paris, France actually *is* Da Vinci’s Mona Lisa, and can be verified as such by art historians. It is objectively authentic. The subjective and constructive element of authenticity is different, however. This element can be understood when considering comments such as, “The Mona Lisa didn’t seem like the real Mona Lisa to me. I thought it would be bigger^1^.” This is a subjective assessment of authenticity, one made apart from expert attestations to the object’s provenance, and thus apart from the reality and validity of the object experienced. The subjective and constructive aspect of authenticity is built on individuals’ dreams, images, expectations, preferences, and beliefs (Wang, 1999). Subjective authenticity may exist even in the absence of an objectively authentic object (“Colonial Williamsburg just seemed so real. It looked like 17th-century America.^2^”).

Activity-related authenticity, also called existential authenticity, is a perception of genuineness in the tourist’s feelings and experiences that emerges by engaging in tourist activities (“Walking into the Colosseum made me feel like I had stepped back into ancient Rome”). It is experiential. To briefly summarize, objective authenticity is related to the genuineness of the object or place itself, constructive authenticity refers to the projections made by the viewer about the object or place, and activity-related authenticity is about the viewer’s internal feelings arising from experiencing an object or place. Definitions appear in Table 1.

**Table 1:** Key definitions related to authenticity

Term	References
*Object-related authenticity* – the authenticity of objects such as art, artifacts, or buildings. It has an objective element as well as a subjective and constructive element.	(Handler & Saxton, 1988; Selwyn, 1996; Wang, 1999)
* Objective authenticity* – refers to the authenticity of originals, such as art, artifacts, historical sites, or other tourist attractions.	(Kolar & Zabkar, 2010; Sharpley, 2018; Trilling, 1972; Wang, 1999)
* Constructive authenticity* – refers to the authenticity projected onto toured objects by tourists or tourism producers in terms of their imagery, expectations, preferences, beliefs, powers, etc.	(Wang, 1999), see also (Bruner, 1991; Cohen, 1972; Kolar & Zabkar, 2010; Salamone, 1997; Silver, 1993)
*Activity-related authenticity* - a perception of genuineness in the tourist’s feelings and experiences that emerges by engaging in tourist activities.	(Brown, 1996; Wang, 1999)

Most prior studies of authenticity investigate its impact in the context of real-world objects such as museums, cultural events, various cities, or heritage sites (Castéran & Roederer, 2013; Guttentag, 2010; Li et al., 2016; Loureiro, 2019; Nguyen & Cheung, 2016; Schwan & Dutz, 2020; Wu et al., 2019; T. Zhang et al., 2018). For instance, researchers have examined the impact of tourists’ characteristics, motivations, and place attachment on the perception of authenticity by collecting data from tourists who visited Canyon de Chelly National Monument in Arizona in the USA (Budruk et al., 2008). Others studied the conceptualization of experienced authenticity out of action, belief, and toured place in order to balance the objective and subjective approaches to defining authenticity (Belhassen et al., 2008). A related study of an evangelical pilgrimage highlighted the balanced view among belief, action, and place, and stressed that object-related authenticity is an important component of authenticity. Still others developed a structural equation model to test the relationships between cultural-related motivations, object-based and existential authenticity, engagement and loyalty (Bryce et al., 2015). Empirical findings indicate that authenticity influences satisfaction (Cho, 2012; Wu et al., 2019). Specific calls for research have been issued, identifying a need to complement this research conducted at heritage sites with research at non-heritage sites (Beck et al., 2019).

These prior studies investigate the influence of authenticity on the physical objects directly, not on virtual objects using VR technologies. It is possible, however, to extend the concept of authenticity to VR. Given that visitors consider replications of museum objects to be authentic if original objects are fragile or hard to be displayed in the museum, and also given that modifications or improvements to objects could be considered as legitimate substitutes and therefore also authentic (Schwan & Dutz, 2020), this begs the question of whether VR reproductions can also be considered authentic. Given that VR has become a popular technology to enhance tourism experiences – both as a supplementary as well as a substitutional tool – the investigation of authenticity can be extended to the newer, emerging context of VR.

Extending the concept of authenticity into VR presents two interesting theoretical issues. First, do VR experiences generate strong perceptions of object-related and activity-related authenticity in both heritage and non-heritage sites? While object-related authenticity and activity-related authenticity have been studied in various locations and places (Beck et al., 2019), many prior studies of authenticity were conducted in the context of heritage tourism sites because tourists’ satisfaction is determined by the activity or experience in the corresponding heritage site. Following the same rationale, it is worthwhile to study the relationship between activity-related authenticity and VR heritage sites whether to investigate the same findings hold. Likewise, it is possible to extend the same question to non-heritage sites, to investigate whether object-related authenticity or activity-related authenticity is an important influence on satisfaction with VR. That is, the role of authenticity can be investigated with respect to two different types of VR objects: heritage VR tourism sites and non-heritage VR tourism sites.

Second, when integrating the concept of authenticity into VR studies, related questions arise regarding how object-related authenticity and activity-related authenticity are linked to other variables that have proven to be important factors in VR and information systems adoption research. For instance, does the aforementioned concept of presence link in any way to the perceptions of object-related and activity-related authenticity in a VR experience? Does system quality link to object-related authenticity, activity-related authenticity, and satisfaction? It is to these very relationships that we now turn as we present our research model and hypotheses.

## Hypothesis Development

In light of the aforementioned theory and literature, this paper seeks to integrate theoretical principles of authenticity (Brown, 1996; Kolar & Zabkar, 2010; Wang, 1999) with features that have been shown to be necessary for successful adoption of other information systems: system quality and presence, which lead to satisfaction with the system (Guttentag, 2010). We propose theoretical applications of this prior research in the emerging context of VR. The model is novel not only for integrating adoption-related constructs with authenticity, but also because it will examine these relationships in both heritage and non-heritage sites.

**Fig. 1:**
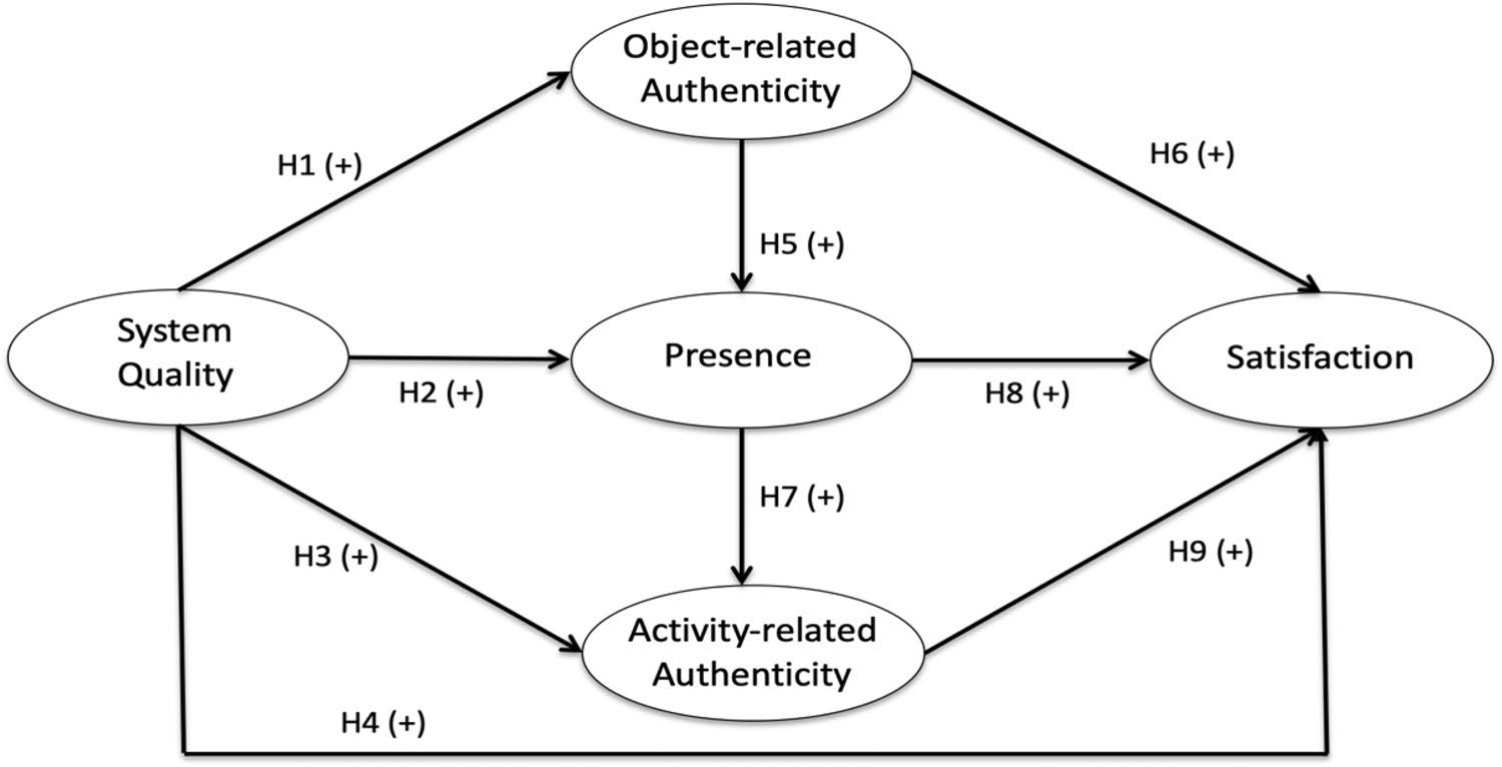
Research model

Specifically, this study examines the relationships among five different variables: system quality, presence, object-related authenticity, activity-related authenticity, and satisfaction. System quality and presence are included based on prior studies of VR. Object-related authenticity and activity-related authenticity are included as major research subjects of this study. In total, nine hypotheses are proposed with respect to heritage sites and non-heritage sites. Finally, the results from heritage sites will be compared to those from non-heritage sites if any differences exist. Figure 1 presents the research model.

### System Quality

System quality has been used to refer to a system’s ease of use, reliability, flexibility, convenience, and functionality (Jung et al., 2016; Tussyadiah et al., 2016), and regarding VR, has been described as affecting the overall successfulness of VR to offer a version of reality (Guttentag, 2010).

While system quality is clearly an important variable in many prior research studies, including those that investigate VR, the impact of system quality on users’ perception of authenticity has not been thoroughly explored (Mura et al., 2017). Since system quality impacts how users interact and gather information from the virtual experience (Dinh et al., 1999), the ability of users to judge the authenticity of what they are viewing is likely to be influenced by system quality. Having a high level of system quality can allow users to better interact with and learn about the items displayed (Guttentag, 2010), increasing the likelihood of perceiving the displays as authentic, leading to object-related authenticity.

In the corporeal world, high-quality replicas are perceived as acceptable at heritage sites, as long as they are clearly identified as being replicas (Schwan & Dutz, 2020) that are true to the original form (Hampp & Schwan, 2015). While the existence of this relationship in the virtual world is unknown, it seems likely that high system quality is necessary to provide a suitable replication of the corporeal world to maintain perceptions of authenticity. We therefore hypothesize,

- Hypothesis 1. System quality is positively associated with object-related authenticity.

The quality of a VR system has been frequently reported to impact the user’s sense of presence while using the system (Baños et al., 2012; Beck et al., 2019; Desai et al., 2014; Dinh et al., 1999; Gutiérrez Alonso et al., 2008; Orru et al., 2019; Slater & Wilbur, 1997). In cases where system quality was found wanting, users reported feeling less immersed in the experience (Cadet & Chainay, 2020).

On the other hand, improved system quality can allow users to feel more present and connected to the site (Baños et al., 2012; Beck et al., 2019, 2019, 2019; Desai et al., 2014; Dinh et al., 1999; Gutiérrez Alonso et al., 2008; Orru et al., 2019; Slater & Wilbur, 1997). This improved presence is observable through a more emotional response in users (Diemer et al., 2015). This leads to the second hypothesis, which would confirm foregoing research, and states

- Hypothesis 2. System quality is positively associated with presence.

Given that system quality has been associated with providing a greater sense of presence and a more immersive experience for users (Beck et al., 2019), it seems plausible that system quality can also affect perceptions of activity-related authenticity. A high degree of system quality will allow users to focus more on the experience with fewer distractions, such as those that arise from poorly functioning software (Cadet & Chainay, 2020). Without such distractions, users can become more immersed in the VR experience that is offered (Beck et al., 2019).

By providing users with a more immersive and fulfilling experience, users may perceive the experience as more genuine because of a more engaging interaction (Moscardo, 2009). Formally, we state

- Hypothesis 3. System quality is positively associated with activity-related authenticity.

System quality has repeatedly been found to significantly impact users’ perception of satisfaction with the system and the information it presents (Chung et al., 2018; Delone & McLean, 2003; Jung et al., 2016; Oghuma et al., 2016; Orru et al., 2019; Tussyadiah et al., 2018; Wei et al., 2019), without notably differentiating between system or information satisfaction, instead describing the overall experience (see Delone & McLean, 2003; Oghuma et al., 2016; Orru et al., 2019). Delone and McLean (2003) explain that high system quality will relate to greater use and improved user satisfaction. Furthermore, specific features of system quality, such as usefulness and ease of use are positively related to user’s perception of satisfaction with the system being used (Oghuma et al., 2016).

The relationship between system quality and satisfaction could be explained by the ability of a high-quality system to be more immersive and less distracting (Beck et al., 2019; Cadet & Chainay, 2020) which correlates with a greater sense of enjoyment (Tussyadiah et al., 2018). Thus, we state

- Hypothesis 4. System quality is positively associated with satisfaction.

### Object-related Authenticity

In terms of the relationship between object-related authenticity and presence, Sanchez-Vives & Slater (2005) claim that when trying to build a sense of presence in viewers, the realism of a VR display is less important than other traits, such as system quality. Similarly, since VR is better at recreating sites than activities (Guttentag, 2010), and since activities and engagement are related to an improved sense of presence (Beck et al., 2019), efforts to improve perceptions of object-authenticity may not be strongly associated with presence. However, since VR is attempting to recreate a real site and experience, part of this recreation could extend to a perception that the objects being viewed are authentic. In a similar perspective to Moscardo’s (2009) discussion on mindfulness, a sense of presence could, therefore, occur if an object is perceived to be authentic. Researchers have suggested that if users feel a greater sense of authenticity, a stronger sense of presence occurs (Wei et al., 2019). These researchers did not, however, distinguish between the types of authenticity: object-related or activity-related.

The disparity therefore seems to center on the perspective being adopted. System-wise, the methods used to create object-related authenticity may counteract, or at least, not support methods to develop presence (Sanchez-Vives & Slater, 2005); one requires more attention on the displays, while the other requires more involvement and interaction (Beck et al., 2019; Guttentag, 2010). Appearance-wise, however, an object that is perceived as authentic could involve users more because the display is less synthetic, and thus, more realistic. (Sylaiou et al., 2010). Continuing this argument, researchers have suggested that items that were perceived to be inauthentic were an impediment to developing a sense of presence (Orru et al., 2019). The impact of these two perspectives on presence is therefore tested in hypothesis 5, following the appearance-based perspective, assuming objects viewed as authentic will develop a sense of presence in users.

- Hypothesis 5. Object-related authenticity is positively associated with presence.

The appearance argument can be extended to also connect object-related authenticity with satisfaction. Objects perceived to be authentic not only made users feel more present, but also provided greater feelings of enjoyment and satisfaction (Cho, 2012; Sylaiou et al., 2010). Likewise, perceived authenticity was the best predictor of satisfaction at historic sites (Moscardo & Pearce, 1986). While these researchers failed to differentiate between the different types of authenticity (see Cho, 2012), their analysis appears to address object-related authenticity, focusing on the authentic appearance of an object. Therefore, in line with the aforementioned appearance-based argument, hypothesis 6 suggests that if a display is made to look and operate in a realistic manner, users would report greater satisfaction. Formally,

- Hypothesis 6. Object-related authenticity is positively associated with satisfaction.

### Presence

A key aspect of VR is its ability to develop a sense of presence in users (Gutiérrez Alonso et al., 2008), wherein users have a sense of being at the site displayed (Guttentag, 2010; Sanchez-Vives & Slater, 2005).One type of authenticity, activity-related authenticity, refers to participants’ perception of having an authentic experience at the site (Wang, 1999). Therefore, both presence and activity-related authenticity have congruent end goals, namely to focus users’ attention on the experience.

Activity-related authenticity occurs when visitors are able to focus on the experience they have at a site (Wang, 1999). This is a desirable outcome since such focus helps visitors remember their experience and learn from it, while experiencing increased satisfaction, and growing loyalty (Moscardo, 2009; Moscardo & Pearce, 1986). At physical sites, it is possible to facilitate this greater experiential focus by making displays and activities more interactive, relevant, and easy to navigate (Moscardo, 2009). In virtual sites, however, VR systems are less effective at reflecting these types of activities (Guttentag, 2010). Instead, VR systems are intended to develop a sense of presence so users feel more involved at the site (Ijsselsteijn & Riva, 2003; Sanchez-Vives & Slater, 2005). Therefore, it is possible that the sense of presence generated by a VR system facilitates users’ perceptions of activity-related authenticity. It is consequently proposed that

- Hypothesis 7. Presence is positively associated with activity-related authenticity.

Greater immersion and presence with a VR system has also been found to result in greater feelings of satisfaction with the experience overall (Beck et al., 2019; Cadet & Chainay, 2020; Chung et al., 2018; Sylaiou et al., 2010; Wei et al., 2019). Immersion and satisfaction also influence users’ desire to reuse (Wei et al., 2019).

The connection between developing presence and user satisfaction has been reported in research on education, psychology, marketing (Tussyadiah et al., 2018), as well as on destination image (Hyun & O’Keefe, 2012). In one study, a positive connection between developing a sense of presence in viewers and their experience of a virtual theme park ride was demonstrated (Wei et al., 2019). Extant literature exploring experiences in museums suggests that this connection could be due to the greater interactivity offered; where the displays are more interactive and immersive, visitors tend to report improved satisfaction (Moscardo, 2009). The connection between presence and satisfaction is presented as hypothesis 8.

- Hypothesis 8. Presence is positively associated with satisfaction.

### Activity-related Authenticity

The relationship between activity-related authenticity and satisfaction has been debated in extant literature. Several studies (Moscardo & Pearce, 1986; Nguyen & Cheung, 2016) have found a positive relationship between perceptions of authenticity and satisfaction, with others taking a more nuanced stance, reporting a positive relationship between activity-related authenticity and satisfaction (Cho, 2012; Park et al., 2019; H. Zhang et al., 2018).

These outcomes could be explained by tourists who are more actively engaged in the experience report greater awareness, learning, and satisfaction with their experience (Moscardo, 2009). While the consensus supports the positive association between activity-related authenticity and satisfaction (Cho, 2012; Park et al., 2019; T. Zhang et al., 2018) some researchers have found that activity-related authenticity had no impact on perceptions of satisfaction, although it did lead to more positive perceptions of value at heritage sites (S. Lee et al., 2016). Given these mixed findings and the general lack of understanding of VR authenticity on satisfaction, we propose investigation of this relationship and, following the directionality demonstrated in the majority of prior studies, we hypothesize

- Hypothesis 9. Activity-related authenticity is positively associated with satisfaction.

### Heritage Sites vs. Non-heritage Sites

Heritage tourism refers to an “activity by tourists in a space where historic artefacts are presented” (Ghermandi et al., 2020). This could include visits to museums, sites of historical or cultural significance (Moscardo, 2009; Trilling, 1972), art studios, or cultural festivals (Cho, 2012). Non-heritage sites include, usually, more leisure-focused sites such as theme parks (Milman, 2013; Waysdorf & Reijnders, 2018; Wei et al., 2019), landmarks, natural parks (Refsland et al., 1998), or shopping centers, which are not considered to significantly reflect a region’s culture or history.

Literature comparing the adoption of VR at heritage as well as non-heritage sites is scant, although some studies have suggested that VR systems are suitable in both heritage and entertainment sites (Guttentag, 2010). Previous studies have explored perceptions of authenticity at heritage sites (Cho, 2012; S. Lee et al., 2016; Moscardo, 2009; Moscardo & Pearce, 1986; Park et al., 2019; H. Zhang et al., 2018), of VR at heritage sites (Sylaiou et al., 2010), and of VR at non-heritage sites such as theme parks (Wei et al., 2019). Understanding the difference between heritage and non-heritage sites in terms of perceptions of VR and authenticity has added importance since the nature of the site can influence attitudes towards the type of authenticity that is preferred (Reisinger & Steiner, 2006; Wang, 1999), especially given recent addition of heritage and non-heritage sites to VR platforms (Radermecker, 2021).

Literature exploring perceptions of authenticity at non-heritage sites is also under-represented. Given past findings that perceptions of authenticity act as a mediating variable between heritage motives and satisfaction (Nguyen & Cheung, 2016), it becomes valuable to understand the impact of authenticity at non-heritage sites.

Non-heritage sites still rely on offering ‘genuine’ artifacts and experiences, although what is considered as genuine may change. For example, while visiting a museum, perceptions of authenticity are likely to focus on the historical and cultural accuracies of the displays or the ability to offer a historical experience to visitors. At non-heritage sites, such as amusement parks, authenticity may extend to the ability of the park to offer an authentically amusing experience, functioning facilities, or consistent adherence to a theme (Milman, 2013; Waysdorf & Reijnders, 2018).

Connections between VR system quality, object-related authenticity, presence, and activity-related authenticity have not clearly been established in either heritage or non-heritage settings. As VR systems are rolled out across different settings (Radermecker, 2021), it becomes increasingly important to understand how users will assess their experiences; how will their perceptions of system quality, authenticity, presence, and satisfaction vary across sites, and how appropriate are authentic VR tools in heritage and non-heritage settings? Such a comparison, exploring relationships at both heritage and non-heritage sites is a novel contribution to literature. In a heritage context, the omission of authenticity is particularly noteworthy, given the significant role authenticity can play is visitors’ perception of heritage sites (M. J. Kim et al., 2020; Nguyen & Cheung, 2016; Yi et al., 2021). The theoretical framework in Fig. 1 and above discussion, therefore seeks to focus on the potential role authenticity can play in affecting perceptions of satisfaction at VR sites, accounting for features already determined to influence VR site satisfaction and satisfaction with IS adoption, such as system quality and presence (Beck et al., 2019; Sylaiou et al., 2010; Tussyadiah et al., 2016).

In order to fully understand the relationships between VR at heritage and non-heritage sites, each of our hypotheses will be tested at heritage sites as well as non-heritage sites.

## Method

### Sample

For this study, the researchers identified 245 potential participants among four undergraduate and graduate courses at two private, coeducational universities, both with a high proportion of international students, both in the United Arab Emirates. Students were drawn from a broad cross-section of majors. Many courses of study are represented, including business (accounting, economics, finance, information systems, marketing, and management), engineering (civil, industrial, mechanical, and engineering students who have not yet chosen a major), mass communication, psychology, and tourism management. Participation in the survey was voluntary, with extra course credit (approximately 1% of their course grade) offered to encourage not only participation but also serious, thoughtful responses. Participants were informed that their responses to the survey items would be separated from identifying information, thus ensuring that responses would be confidential and anonymous while also enabling course credit. Participants were free to quit the survey and withdraw from the study at any time.

Of the 245 potential participants, researchers received a total of 196 responses (80.00% response rate). Out of 196 responses, only 3 were dropped since they were straight-line responses where the same numbers from the Likert scale questions were selected for each and every item. Thus, 193 responses were valid, complete, and therefore analyzed for this study.

While the use of students as research subjects has been questioned (Gallander Wintre et al., 2001; Sears, 1986), student samples are nevertheless appropriate in many research settings. The use of students is rightly criticized in studies of strategic management, for instance. Telling undergraduate students, “Imagine you are a CEO…” when answering questions about leadership and strategic management will almost certainly not yield valid responses that can be generalized to the target population of C-level executives. Executive MBA students could perhaps provide a reasonable approximation of C-level leaders, but undergraduate students do not have the needed experience or perspective. The crucial question when considering student samples is one of generalizability – do the results generalize to the target population? In this study, the research questions focus on tourists’ experiences using VR. While Dubai360.com is a relatively new system that the majority of respondents have not used before, the student subjects in this study are familiar with the desktops, laptops, and smartphones being used, and thus are not encumbered by technological inexperience. They can assess whether a system is one of sufficient quality or not. They are also familiar with several of the heritage and non-heritage sites and thus can make assessments about authenticity and whether their VR experience provides a sense of presence. Furthermore, students could constitute the target audience of the selected sites given their age, technological awareness, and likelihood to travel. The use of a student sample seems appropriate here. Results should generalize to the population of tech-savvy travelers targeted by the creators of niVR websites with 360-degree views of attractions.

Respondents included 87 males (45.08%) and 106 females (54.92%). The majority have lived in the UAE more than 10 years (137 respondents, 70.98%), with 19 respondents having lived in the UAE between 5–10 years (9.84%), and 37 having lived in the UAE less than 5 years (19.17%). Respondents included 5 freshmen/first-year students (2.59%), 94 sophomores/second-year students (48.70%), 48 juniors/third-year students (24.87%), 44 seniors/fourth-year students (22.79%), and 2 fifth-year or graduate students (1.03%). Descriptive statistics appear in Table 2 below.

**Table 2:** Demographic descriptive statistics (n = 193)

Gender	Frequency	Percentage
Male	87	45.08%
Female	106	54.92%
Total	*193*	*100%*
Length of Residency		
> 10 years	137	70.98%
5–10 years	19	9.84%
< 5 years	37	19.17%
Total	*193*	*100%*
Classification		
Freshman/First-year	5	2.59%
Sophomore/Second-year	94	48.70%
Junior/Third-Year	48	24.87%
Senior/Fourth-Year	44	22.79%
Graduate or Fifth-Year	2	1.03%
Total	*193*	*100%*

Overall, the sample is approximately balanced between males and females. Respondents are familiar with the UAE, with over 80% having lived in the country for five or more years, and thus likely possess some awareness of its tourist attractions, such as the ones mentioned in the survey. They should thus be able to make assessments of the authenticity of the tourist sites that are presented. Additionally, the sample of largely undergraduate students seems appropriate. Such respondents will be in their 20s, and thus generally familiar with the technology needed to navigate the VR site and respond to the survey. Furthermore, respondents of this age represent a key demographic VR site creators are targeting with their content.

### Data Collection

Data was collected during regularly-scheduled class sessions using a Google Forms survey. The VR experience used in this study was a non-immersive VR (niVR) site, Dubai360.com. Of these three types of VR, niVR, siVR, and fiVR, niVR applications are the simplest to create, and are therefore by far the most numerous. Given their prevalence, niVR was selected for this study.

Recent calls for research specifically using niVR sites have gone out (Beck et al., 2019), with researchers emphasizing the need to study niVR sites that use recent technological developments, including 360-degree footage, in tourism contexts. The Dubai360 niVR website was chosen for this study because of its popularity, convenience of use and access, as well as the significant amount of both heritage as well as non-heritage content. Furthermore, no special equipment (such as head-mounted displays), nor special input devices, nor special training, was required for use of the site and participation in the survey.

Researchers gave a 15–20 minute orientation to the Dubai360 VR site, emphasizing the site’s features and functionality, and also showing how to navigate around the site and virtually visit the specified attractions. After the Dubai360 orientation provided by the researchers, participants were then provided a link to either version 1 of the survey, for heritage tourist sites, or version 2 of the survey for non-heritage sites. Before the collection of data, respondents were explained that Dubai360.com is one type of VR sites, but in the questionnaire they were asked to evaluate Dubai360 website without the VR term because the majority of respondents considered it as one of websites they could visit conveniently.

Using a cluster random sample, the four classes were randomly assigned to either version 1 or version 2 to ensure both versions received roughly equal participation. Participants were encouraged to use a desktop or laptop computer for ease of navigation, but could use a smartphone as well.

Both version 1 and version 2 of the survey began with four initial questions about specific tourist attractions. Version 1 featured questions about three specific heritage sites and version 2 about three specific non-heritage sites. Details about each site and screenshots appear in Appendix. To ensure participants were actively utilizing the site and its features, answers to these four initial questions required navigation around and use of the Dubai360 website to gather information. After these initial questions, the main items were presented to assess the research variables, each using a 1–7 Likert scale (see Section 4.3 for details regarding the survey items). Finally, four demographic questions were posed. Participants averaged 36 minutes, 30 seconds to navigate around the site and complete the survey (ranging from 10 minutes, 51 seconds to 1 hour, 39 minutes, and 16 seconds). Of the responses, 92 were for version 1 (heritage sites) and 101 were for version 2 (non-heritage sites).

### Development of Instrument and Measurement Items

This study used constructs with multiple-item scales. Responses were scored on a seven-point Likert scale. Where possible, existing measures validated in prior studies were adopted. Where necessary, items from prior studies were revised carefully to reflect the VR context of the present study. Table 3 shows the specific items used in this study. System quality, presence, and satisfaction were directly adopted from prior studies. Object-related authenticity and activity-related authenticity were revised from prior studies that investigated authenticity in offline contexts.

**Table 3:** Measurement Items

Constructs	Measurement Items	Source
System quality (SYSQ)	I think the Dubai360 website is • SYSQ1 - Easy to use. • SYSQ2 - Fast. • SYSQ3 - Convenient to use. • SYSQ4 - Easy to navigate.	(H.-C. Kim & Hyun 2016)
Presence (PRES)	While visiting the recommended attractions on the Dubai360 website, • PRES1 - I felt like that I have actually been there. • PRES2 - It seemed as if I actually took part in sightseeing. • PRES3 - It was as if my true location has shifted to the virtual environment. • PRES4 - I felt as if I was physically present in the virtual environment.	(Kang & Gretzel, 2012)
Object- related authenticity (OBAU)	While visiting the recommended attractions on the Dubai360 website, • OBAU1 - It looked like real. • OBAU2 - It was as accurate as the real attractions. • OBAU3 - It showed me the genuine features of the attractions. • OBAU4 - I was able to figure out the real features of the attractions. • OBAU5 - It accurately reproduced the real object virtually.	Castéran & Roederer, 2013; Lin & Liu, 2018; Wu et al., 2019; Zhou et al., 2015)
Activity- related authenticity (ACAU)	While visiting the recommended attractions on the Dubai360 website, • ACAU1 - I feel like I truly experienced these attractions. • ACAU2 - Watching the site made me connected to these attractions. • ACAU3 - I was immersed in the atmosphere of the attractions. • ACAU4 - I was able to escape from my daily life.	(Kolar & Zabkar, 2010; Zhou et al., 2015)
Satisfaction (SAT)	How do you feel about your overall experience of visiting the recommended attraction? • SAT1 - Satisfied. • SAT2 - Pleased. • SAT3 - Content.	(Wu et al., 2019)

The possible presence of common method bias was examined before conducting the measurement assessment. Self-reported data collected from the same person at one time could yield unintended correlations that contaminate data obtained from that source (Malhotra et al., 2006). The risk of common method variance was tested in two statistical analysis: Harman’s one factor test (Podsakoff et al., 2003), and Lindell and Whitney’s (Lindell & Whitney, 2001) marker variable test. For Harman’s one-factor test, if the total variance extracted by one factor is higher than 50%, it is considered that the sample has a problem of common method bias. For this study, no such single factor emerged and the first factor explained only 20.05% of total 73.25% variance. For Lindell and Whitney’s marker variable test, a theoretically unrelated marker variable is adopted to test the correlations among the model’s principal constructs. We used the variable of security awareness, and the average correlation of the study’s research variables with it was low and insignificant. Security awareness was measured by three items: I discuss with friends and people around me security issues of internet, I read about the problems of malicious software including internets users’ computers, and I am aware of the spyware problems and consequences (Dinev & Hu, 2007). Thus, we concluded that common method bias has not influenced the results in this study.

## Results and Analysis

This study adopted the Partial Least Squares (PLS) method to perform an evaluation of the measurement model as well as the structural model. The PLS structural equation modeling method (PLS-SEM) is more suitable for theory building and theory testing (Hair et al., 2017). Given that PLS-SEM is more prediction-oriented, PLS is considered to be appropriate since this study is one of the first attempts to investigate the effect of authenticity on VR satisfaction.

### Measurement Assessment

The internal consistency of constructs was assessed by examining Cronbach’s alpha (α) and composite reliability (CR). The internal consistency requirements (Lindell & Whitney, 2001; Podsakoff et al., 2003) are satisfied when scores of both tests exceed 0.7. Results in Table 4 indicate that all Cronbach’s α values are higher than 0.7 and CRs values range from 0.70 to 0.95.

**Table 4:** Item cross-loadings and internal consistency measures

ITEM	SYSQ	PRES	OBAU	ACAU	SAT	Alpha	C.R.	AVE
SYSQ1	**0.808**	0.256	0.260	0.295	0.379	0.800	0.817	0.698
SYSQ2	**0.702**	0.254	0.268	0.272	0.381			
SYSQ3	**0.843**	0.391	0.355	0.341	0.466			
SYSQ4	**0.829**	0.341	0.260	0.313	0.318			
PRES1	0.297	**0.827**	0.406	0.569	0.328	0.893	0.896	0.757
PRES2	0.362	**0.871**	0.420	0.561	0.333			
PRES3	0.375	**0.887**	0.346	0.694	0.369			
PRES4	0.353	**0.894**	0.334	0.678	0.345			
OBAU1	0.263	0.381	**0.807**	0.390	0.232	0.838	0.848	0.606
OBAU2	0.220	0.333	**0.783**	0.276	0.195			
OBAU3	0.306	0.357	**0.830**	0.425	0.439			
OBAU4	0.316	0.338	**0.728**	0.375	0.339			
OBAU5	0.292	0.252	**0.740**	0.393	0.246			
ACAU1	0.343	0.691	0.449	**0.868**	0.380	0.878	0.916	0.733
ACAU2	0.328	0.607	0.425	**0.885**	0.377			
ACAU3	0.359	0.600	0.461	**0.862**	0.445			
ACAU4	0.293	0.566	0.306	**0.807**	0.293			
SAT1	0.454	0.302	0.301	0.328	**0.824**	0.783	0.790	0.698
SAT2	0.469	0.349	0.365	0.406	**0.894**			
SAT3	0.308	0.338	0.347	0.363	**0.786**			

SYSQ: system quality, PRES: presence, OBAU: object-related authenticity, ACAU: activity-related authenticity, SAT: satisfaction

Bold face items signify variable cross-loading measures for a single construct

For convergent validity, the average variance extracted (AVE) should be higher than 0.5 and factor loading scores of each construct should be higher than 0.7 (Hair et al., 2017). Table 5 shows that all constructs satisfy the requirements of convergent validity, with AVE scores higher than 0.6 (average value = 0.695) and factor loadings ranging from 0.7 to 0.9.

**Table 5:** Discriminant validity

	SYSQ	PRES	OBAU	ACAU	SAT
SYSQ	**0.835**				
PRES	0.393	**0.870**			
OBAU	0.351	0.425	**0.778**		
ACAU	0.383	0.715	0.461	**0.856**	
SAT	0.486	0.395	0.379	0.431	**0.835**

Bold face items on the diagonal are the square root of AVE. SYSQ: system quality, PRES: presence, OBAU: object-related authenticity, ACAU: activity-related authenticity, SAT: satisfaction

Discriminant validity is confirmed since the correlation between pairs of constructs is lower than 0.9 and the square root of AVE is greater than its correlation estimates, and the cross-loadings of factor scores are higher in the corresponding construct than loadings in other constructs (Table 5). Therefore, we conclude that the three criteria of discriminant validity are satisfied.

### Structural Model Assessment

**Fig. 2:**
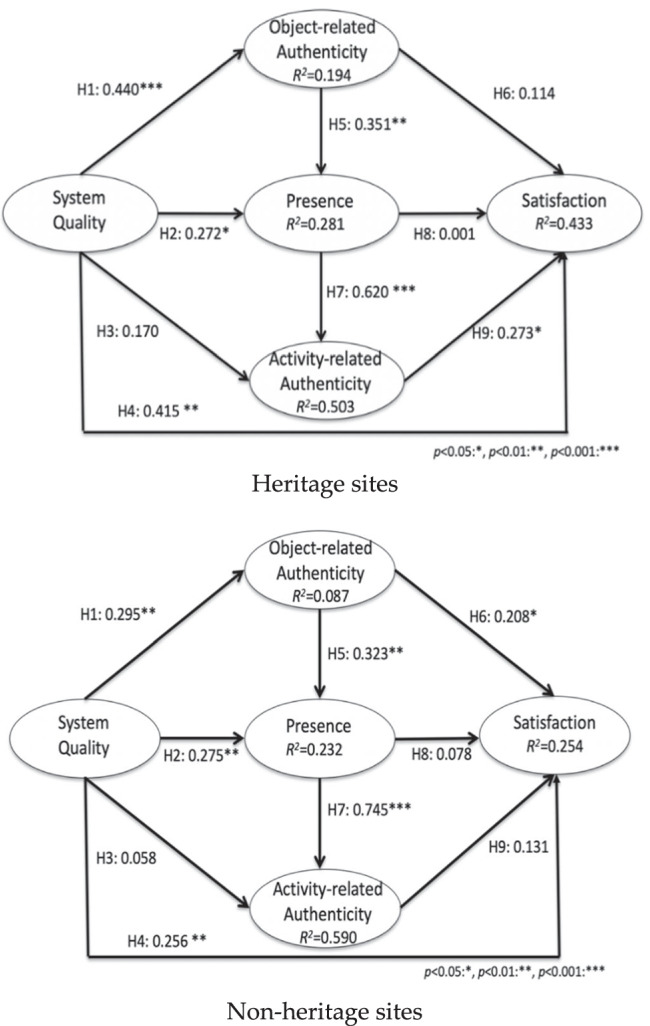
Results of the structural model tests

Smart PLS was used to estimate the structural model. A bootstrapping procedure with resampling of 500 subsamples was used to determine the statistical significance of estimates. Figure 2 and Table 6 show the results for two different types of sites: heritage and non-heritage sites. For the two different types of sites, research models and hypotheses are proposed with the same numbering and in the same order. This study compares the differences between them.

**Table 6:** Results of Hypotheses and Multi-Group Analysis

Hypotheses No.	Heritage sites *β(p)*: Test Result	Non-heritage sites *β(p): Test* Result	Multi-group analysis *p*-value (heritage vs. non-heritage)
H1	0.440 (0.000)	0.295 (0.005)	0.474
H2	0.272 (0.032)	0.275 (0.011)	0.454
H3	0.170 (0.078): rejected	0.058 (0.380): rejected	0.464
H4	0.415 (0.003)	0.256 (0.021)	0.478
H5	0.351 (0.002)	0.323 (0.002)	0.364
H6	0.114 (0.378): rejected	0.208 (0.037)	0.048
H7	0.620 (0.000)	0.745 (0.000)	0.264
H8	0.001 (0.996): rejected	0.078 (0.573): rejected	0.716
H9	0.273 (0.016)	0.131 (0.412): rejected	0.042

For heritage sites, results indicate that system quality is positively associated with object-related authenticity (H1: β = 0.440, *p* = 0.000), presence (H2: β = 0.272, *p* = 0.032), and satisfaction (H4: β = 0.415, *p* = 0.003), but not with activity-related authenticity (H3: β = 0.170, *p* = 0.078). Object-related authenticity is positively associated with presence (H5: β = 0.351, *p* = 0.002) which is, in turn, positively associated with activity-related authenticity (H7: β = 0.620, *p* = 0.000), but does not have a positive relationship with satisfaction (H6: β = 0.114, *p* = 0.378). Therefore, we find that satisfaction is positively associated with system quality (H4) and activity-related authenticity (H9: β = 0.273, *p* = 0.016). Presence (H8: β = 0.001, *p* = 0.996) and object-related authenticity (H6) do not have positive relationships with satisfaction.

For non-heritage sites, results indicate that system quality is positively associated with object-related authenticity (H1: β = 0.295, *p* = 0.005), presence (H2: β = 0.275, *p* = 0.011), and satisfaction (H4: β = 0.256, *p* = 0.021), but not with activity-related authenticity (H3: β = 0.058, *p* = 0.380). Object-related authenticity is positively associated with presence (H5: β = 0.323, *p* = 0.002) which is, in turn positively associated with activity-related authenticity (H7: β = 0.745, *p* = 0.000) but not with satisfaction (H8: β = 0.078, *p* = 0.573). Therefore, we find that satisfaction is positively associated with two variables of system quality (H4) and object-related authenticity (H6: β = 0.208, *p* = 0.037), not with presence (H8) or activity-related authenticity (H9: β = 0.131, *p* = 0.412).

Thus, the results from tests of H1, H2, H3, H4, H5, H7 and H8 are consistent between heritage sites and non-heritage sites (see Fig. 2). Only H6 and H9 differ. Regarding the dependent variable of satisfaction, results differ at heritage and non-heritage sites. At heritage sites, object-related authenticity is not a statistically significant predictor of satisfaction, but activity-related authenticity is. At non-heritage sites, the converse is seen. Object-related authenticity is positively and significantly related to satisfaction, but the link from activity-related authenticity to satisfaction is not statistically significant. This result is confirmed by the multi-group analysis using Smart PLS, as shown in the 3rd column in Table 6. The result indicates that the predictors between heritage and non-heritage sites are statistically different in H6 and H9 at the 5% level of significance. This difference between the predictors for satisfaction is the major distinction between heritage sites and non-heritage sites. Explanations and implications of this will be described in Section 6.

The R^2^ values of the model for heritage sites were 0.194, 0.281, 0.503, and 0.433 for objective authenticity, presence, activity-related authenticity, and satisfaction, respectively. The R^2^ values of the model for non-heritage sites were 0.087, 0.232, 0.590, and 0.254 for objectively authenticity, presence, activity-related authenticity, and satisfaction, respectively. In both models, over 50% of the variation in activity-related authenticity is explained by presence. Overall, the explanatory power of the model for heritage sites is higher than for non-heritage sites since 43.3% of the variability of satisfaction is explained in heritage sites and only 25.4% of the variability is explained in non-heritage sites. Even though 43.3% of R^2^ value in heritage sites is acceptable, this result obviously implies that other exogeneous variables exist that explain the variability in satisfaction. This was an expected outcome since the model did exclude any other VR variables such as perceived usefulness or enjoyment, focusing only on the effect of authenticity.

### Summary of Results

We found that authenticity is an important variable affecting both users’ perceptions of presence as well as users’ satisfaction with VR. Even though the issue of the authenticity of VR tourism was first raised over a decade ago, empirical studies have rarely been conducted (Beck et al., 2019; Guttentag, 2010). We confirm the idea that realistic objects serve as a way to enhance tourism and connect a virtual experience to a real one (Refsland et al., 1998). We also confirm the finding that realistic objects enhance presence in VR (Tussyadiah et al., 2018).

Furthermore, users’ perceptions regarding authenticity were proposed as determinants of acceptance of VR as a substitute for a real-world tourism experience (Guttentag, 2010). It was found that at heritage sites, system quality influenced satisfaction directly, and indirectly by positively affecting perceptions of object-based authenticity which, in turn provided a sense of presence which then created perceptions of activity-based authenticity. Perceptions of activity-based authenticity were then related to satisfaction. At non-heritage sites, a similar relationship was noted, with some minor variations. System quality also directly influenced perceptions of satisfaction. However, satisfaction was related to object-based authenticity.

As for presence, it has been considered one of the important factors affecting the performance of VR (Tussyadiah et al., 2018). This study’s result is partially consistent with prior studies, with presence only indirectly related to satisfaction with VR. Specifically, at heritage sites, presence is indirectly related to satisfaction by way of activity-related authenticity. It is not associated with satisfaction at all at non-heritage sites. Presence is not a direct determinant of VR satisfaction and the role of presence is different depending on the type of destinations such as heritage and non-heritage sites. This finding needs to be further investigated to better understand the specific role of presence with respect to various types of destinations and VR content.

Perhaps most interestingly, results indicate that satisfaction is affected by different factors depending on the type of site being visited. Specifically, satisfaction with VR experiences at heritage sites is driven by activity-related authenticity, while satisfaction at non-heritage sites is driven by object-related authenticity. This result is somewhat paradoxical, given that some may assume that visitors to heritage sites focus on objects (particularly objects of cultural and historical significance) while visitors to non-heritage sites focus on activities.

## Discussion

### Theoretical Implications

Our interpretation of these results is that VR users’ perceptions of heritage sites are shaped by more than simply the historical and cultural objects at the site. They are influenced by the atmospherics of the experience as well. For a heritage site to be truly perceived as such, it must be perceived as distinct and different and as an escape from the tourist’s everyday life. The tourists should perceive that they have been transported to another point in time. In the research subjects’ experience in this study, for instance, the Al Fahidi neighborhood of Dubai is a satisfying experience only if it “feels” like old Dubai with winding alleyways, stone pavements, and coral-brick houses. It should be a world apart from the glass, concrete, and steel metropolis that they experience every day. The activity-related authenticity that is experienced internally and psychologically is important, even more so than the externally and subjectively-experienced object-related authenticity. Thus, the experiential activity (not just the objects) creates satisfaction at heritage sites. Furthermore, while users may accept the use of replicated objects at heritage sites (Schwan & Dutz, 2020), the VR-created virtual environment with its replicated objects may not sufficient to promote satisfaction without an accompanying experiential component.

In the case of non-heritage sites, this study found that visitors to non-heritage VR experiences expect accurate simulations of what they have pictured in their minds. This result needs to be carefully interpreted. In this study, heritage VR sites provided users various historical and cultural content and let them experience the destination virtually during navigation. In contrast, non-heritage sites are different in that there is neither historical nor cultural content. Rather, the major content is the artifacts of a modern city and the interior of luxurious hotel in Dubai. For this reason, it is possible to interpret that users were more interested in the realistic presentation and originality of human artifacts without the feeling of experiencing the destination.

However, this interpretation needs to be investigated further to clarify the difference between heritage sites and non-heritage sites. For instance, what if non-heritage VR sites include dynamic content such as a roller-coaster in an amusement park? Likewise, what if heritage VR sites include more dynamic features such as riding a camel around the Egyptian Pyramids? How will object-related authenticity’s effects on satisfaction and other variables be different from activity-related authenticity’s effects under the different contexts of heritage and non-heritages sites? To understand and interpret the results accurately, further studies need to be conducted that include different types of destination.

Ultimately, our research contributes to work on authenticity, extending the application and discussion of this important theoretical construct to VR contexts, affirming its importance beyond the physical world.

### Practical Implications

The practical implications of our study are first, for those who operate tourism sites and are either currently using or may be considering a VR experience, and second, for the creators/programmers of VR experiences. Both groups should note that quality is vital and is a driver of object-related authenticity; this is true for heritage and non-heritage sites. Quality, in terms of ease of use, speed, reliability, convenience, and navigation, must be prioritized over other design considerations if the objects of the VR tourism experience are to be perceived as authentic. Quality is also essential to create the feeling of presence, of “being there”, at the VR-simulated site. Tour site operators and VR creators should focus on quality to enable users to feel a connection to the VR-simulated destination, to escape from their daily lives, and to experience as much of the atmosphere and ambiance of the destination as possible.

Regarding distinctions between heritage and non-heritage sites, we remind practitioners working with heritage sites to emphasize the activity-related authenticity of their VR experience. While heritage sites may have a proclivity for emphasizing authentic objects, our findings reveal the importance of activities, and their associated feelings and experiences. Equally, practitioners working with non-heritage sites should clearly focus on object-related authenticity. The objects in their VR simulation must appear authentic, facilitating visitors’ projection of their expectations and beliefs about the non-heritage site onto the VR representation of it. Furthermore, practitioners associated with such sites are encouraged to fully investigate potential visitors’ expectations to ensure that their VR simulations do not disappoint, eroding the brand of the tourist site and discouraging VR visitors from visiting in the real world.

### Limitations and Future Research

As with all research studies, this one has limitations. The first is what may initially seem to be a small sample size. In the era of big data, sample sizes are growing larger and larger (Einav & Levin, 2014) and *n* = 193 may be initially perceived as small. Nevertheless, this sample gives adequate statistical power to investigate the hypotheses. Even though PLS can accommodate the problem of small samples, following the 10 times rule of the minimum sample size (Barclay et al., 1995), the minimum required sample size of this study is 50, which implies that the sample size of heritage sites as well as non-heritage sites is more than adequate to produce reliable statistical results.

Second, this study makes use of a student sample. While its use is justifiable and the sample size is suitable for the reasons stated in Section 4, future research may be conducted to extend these results by collecting data from a broader sample.

Third, this study makes use of only non-immersive VR (niVR). The 360-degree view provided on a website is clearly distinct from semi-immersive (siVR) and fully-immersive (fiVR) experiences. Users’ assessments of authenticity and presence may differ when using siVR and fiVR. In view of the results here, and considering the characteristics of siVR and fiVR, we propose that more-immersive VR experiences should lead to stronger assessment of object-related authenticity as well as activity-related authenticity. High-quality siVR and niVR, as well as high levels of object-related authenticity should also lead to a greater sense of presence. In sum, the links between variables in our research model should only be strengthened in research with more immersive types of VR. These suppositions are an obvious avenue for future research.

Fourth, while the length of our respondents’ time in Dubai was not statistically significant in this study, we conjecture that the “familiarity” of tourists with a heritage or non-heritage site may influence their perceptions of authenticity and their satisfaction with the VR experience. Future research may include ways to assess familiarity. Measurement may include the number or length of prior visits to a site, the tourist’s level of exposure to similar sites, or other measures.

Fifth and finally, future study may classify the type of VR destinations in detail. This study classified VR sites only as heritage and non-heritage sites. For both, it is possible to classify further based on the degree of experiential features and the degree of accuracy of objects. For instance, it seems plausible that the accuracy of an object will be more important to VR users for heritage sites with fixed objects such as items at a museum than to VR users for heritage sites with more-active experiences such as a virtual tour walking through a heritage site. In this case, it is possible that object-related authenticity could be more influential on satisfaction than activity-related authenticity in heritage sites. Depending of the types of objects, the exact properties of authenticity may vary. These relationships bear investigating and should be considered in the future.

## Conclusions

The use of VR technology is becoming more popular over time and across different areas of life (Hyun & O’Keefe, 2012). As VR begins to find applications within the domains of tourism, and notably heritage destinations, it becomes more important to understand the relationship between VR and authenticity. It is important to understand how authenticity is affected because of the benefits providing authentic objects and experiences; improved satisfaction (Moscardo & Pearce, 1986; Sylaiou et al., 2010; Wu et al., 2019; H. Zhang et al., 2018), learning (Moscardo & Pearce, 1986), and loyalty (Park et al., 2019) being among them. While research on authenticity is common at heritage sites (Guttentag, 2010; Wu et al., 2019; T. Zhang et al., 2018), the lack of consideration at and comparison with non-heritage sites (Milman, 2013; Waysdorf & Reijnders, 2018) is noteworthy. Furthermore, the lack of authenticity in VR research is clear and surprising; VR is meant to create a virtual environment (Guttentag, 2010) in which the user feels transported to the site (Hobson & Williams, 1995), yet research has yet to fully understand how users perceived the authenticity – genuineness – of their viewings or experiences (Mura et al., 2017). This research, therefore, set out to explore how visitors perceive the authenticity of virtual tours of cultural heritage and non-heritage sites?

In this study, the main difference between heritage and non-heritage sites was between which variables indirectly influenced satisfaction. At heritage sites, activity-based authenticity affected satisfaction, after being affected by presence which was in turn affected by object-based authenticity. While at non-heritage sites, the same relationship was present between object-based authenticity, presence, and activity-based authenticity, but this time, it was object-based and not activity-based authenticity which related to satisfaction.

The results suggest that if tourism sites are planning on implementing VR, it is imperative that the system be of high quality to allow the site to provide an authentic and satisfying experience. At heritage sites, it is important to understand that VR contributes to the user’s experience. Therefore, while providing realistic displays will not directly impact satisfaction, it will provide users with a more authentic experience which will relate to satisfaction. At non-heritage sites, it is important that the displays are realistic and objective features as this has a direct impact upon users’ satisfaction. We look forward to the application and implementation of these findings, as well as to future related research that will extend and enlarge up on them.

## Figures and Tables

**Table 7:**
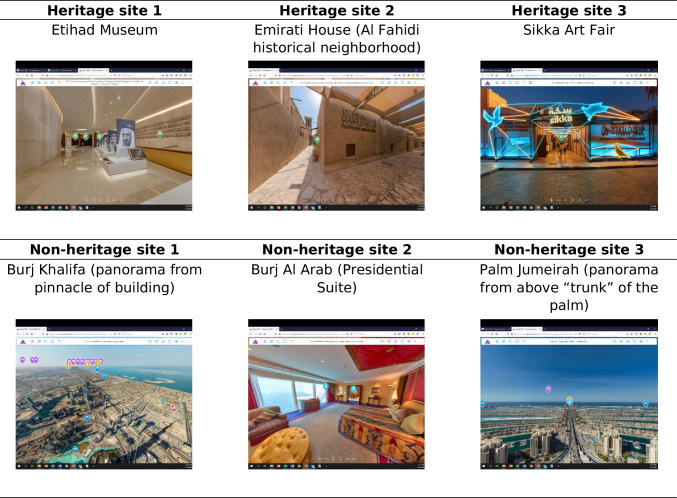
Heritage and non-heritage sites for survey versions 1 and 2

## Appendix

- As shown in Table 7, heritage sites included:
- the Etihad Museum (museum focusing on formation of the UAE as a modern nation),
- the Sikka Art Fair (handicrafts market plus art installations in historical neighborhood), and.
- the Al Fahidi Historical Neighborhood’s traditional Emirati House (preserved/restored home with historical exhibits and interpretive information).
- These three specific heritage sites were chosen based on their cultural and historical significance to the history of the UAE. Images of each heritage and non-heritage site appear in Appendix Table 7.

As shown in Table 7, non-heritage sites included:

- the Burj Khalifa (the tallest building in the world),
- the Burj Al Arab hotel (Dubai’s sail-shaped “7-star” luxury hotel), and.
- the Palm Jumeirah (palm tree-shaped artificial islands).
- Site popularity was the key criterion for choosing these sites.

Table 7

## Footnotes

1 Visitors to the Louvre are often surprised by the size of the Mona Lisa, 77 cm × 53 cm (30 in × 21 in). Many find it smaller than expected. See, for instance visitor comments at https://www.tripadvisor.com/ShowUserReviews-g187147-d188757-r451793284-Louvre_Museum-Paris_Ile_de_France.html

2 3^3^ Colonial Williamsburg is a 300-acre historical area in Virginia, USA with several hundred restored and re-created buildings from the17th ththrough 19th centuries, depicting life in colonial America. See https://www.colonialwilliamsburg.org/

## References

[CR1] Akhtar N, Khan N, Mahroof Khan M, Ashraf S, Hashmi MS, Khan MM, Hishan SS (2021). Post-COVID 19 tourism: Will digital tourism replace mass tourism?. Sustainability.

[CR2] Baños RM, Etchemendy E, Castilla D, García-Palacios A, Quero S, Botella C (2012). Positive mood induction procedures for virtual environments designed for elderly people. Interacting with Computers.

[CR3] Barclay, D. W., Thompson, R., & Higgins, C. (1995). The partial least squares (PLS) approach to causal modeling: Personal computer use as an illustration. *Technology Studies*, *2*(2)

[CR4] Bardi, J. (2019). *What is Virtual Reality? VR Definition and Examples*. Marxent. https://www.marxentlabs.com/what-is-virtual-reality/. Accessed 15 Feb 2021.

[CR5] Beck J, Rainoldi M, Egger R (2019). Virtual reality in tourism: A state-of-the-art review. Tourism Review.

[CR6] Bednar PM, Welch C (2020). Socio-technical perspectives on smart working: Creating meaningful and sustainable systems. Information Systems Frontiers.

[CR7] Belhassen Y, Caton K, Stewart WP (2008). The search for authenticity in the pilgrim experience. Annals of Tourism Research.

[CR8] Brown, D. (1996). Genuine fakes. In T. Selwyn (Ed.), *The tourist image: Myths and myth making in tourism* (pp. 33–47). Wiley

[CR9] Bruner EM (1991). Transformation of self in tourism. Annals of Tourism Research.

[CR10] Bryce D, Curran R, O’Gorman K, Taheri B (2015). Visitors’ engagement and authenticity: Japanese heritage consumption. Tourism Management.

[CR11] Budruk M, White DD, Wodrich JA, Van Riper CJ (2008). Connecting visitors to people and place: Visitors’ perceptions of authenticity at Canyon de Chelly National Monument, Arizona. Journal of Heritage Tourism.

[CR12] Cadet LB, Chainay H (2020). Memory of virtual experiences: Role of immersion, emotion and sense of presence. International Journal of Human-Computer Studies.

[CR13] Castéran H, Roederer C (2013). Does authenticity really affect behavior? The case of the Strasbourg Christmas Market. Tourism Management.

[CR14] Cheong R (1995). The virtual threat to travel and tourism. Tourism Management.

[CR15] Cho M (2012). A study of authenticity in traditional Korean folk villages. International Journal of Hospitality & Tourism Administration.

[CR16] Chung N, Lee H, Kim JY, Koo C (2018). The role of augmented reality for experience-influenced environments: The case of cultural heritage tourism in Korea. Journal of Travel Research.

[CR17] Cohen E (1972). Toward a sociology of international tourism. Social Research.

[CR18] Delone WH, McLean ER (2003). The DeLone and McLean model of information systems success: A Ten-year update. Journal of Management Information Systems.

[CR19] Desai R, Desai P, Deepak NP, Mehta K (2014). A review paper on Oculus Rift-A virtual reality headset. International Journal of Engineering Trends and Technology.

[CR20] Diemer, J., Alpers, G. W., Peperkorn, H. M., Shiban, Y., & Mühlberger, A. (2015). The impact of perception and presence on emotional reactions: A review of research in virtual reality. *Frontiers in Psychology*, *6*. 10.3389/fpsyg.2015.00026 10.3389/fpsyg.2015.00026 PMC4311610 25688218

[CR21] Dinev T, Hu Q (2007). The centrality of awareness in the formation of user behavioral intention toward protective information technologies. Journal of the Association for Information Systems.

[CR22] Dinh, H. Q., Walker, N., Hodges, L. F., Song, C., & Kobayashi, A. (1999). Evaluating the importance of multi-sensory input on memory and the sense of presence in virtual environments. *Proceedings IEEE Virtual Reality (Cat. No. 99CB36316)*, 222–228. 10.1109/VR.1999.756955

[CR23] Domínguez-Quintero AM, González-Rodríguez MR, Paddison B (2020). The mediating role of experience quality on authenticity and satisfaction in the context of cultural-heritage tourism. Current Issues in Tourism.

[CR24] Duan X, Chan C, Marafa LM (2019). Does authenticity exist in cultural theme parks? A case study of Millennium City Park in Henan, China. Journal of Tourism and Cultural Change.

[CR25] Einav L, Levin J (2014). Economics in the age of big data. Science.

[CR26] Gallander Wintre M, North C, Sugar LA (2001). Psychologists’ response to criticisms about research based on undergraduate participants: A developmental perspective. Canadian Psychology/Psychologie Canadienne.

[CR27] Ghermandi, A., Camacho-Valdez, V., & Trejo-Espinosa, H. (2020). Social media-based analysis of cultural ecosystem services and heritage tourism in a coastal region of Mexico. *Tourism Management*, *104002*. 10.1016/j.tourman.2019.104002

[CR28] Gittens CL (2021). Remote HRI: A methodology for maintaining COVID-19 physical distancing and human interaction requirements in HRI studies. Information Systems Frontiers.

[CR29] Gutiérrez Alonso, M. A., Vexo, F., & Thalmann, D. (2008). *Stepping into virtual reality*. Springer

[CR30] Guttentag DA (2010). Virtual reality: Applications and implications for tourism. Tourism Management.

[CR31] Hair, J. F., Hult, G. T. M., Ringle, C., & Sarstdet, M. (Eds.). (2017). *A primer on partial least squares structural equation modeling (PLS-SEM)* (2nd Ed). Sage

[CR32] Hampp C, Schwan S (2015). The role of authentic objects in museums of the history of science and technology: Findings from a visitor study. International Journal of Science Education, Part B.

[CR33] Handler R, Saxton W (1988). Dyssimulation: Reflexivity, narrative, and the quest for authenticity in “living history”. Cultural Anthropology.

[CR34] Hobson PJS, Williams AP (1995). Virtual reality: A new horizon for the tourism industry. Journal of Vacation Marketing.

[CR35] Huang TJ, Chi SC, Lawler JJ (2005). The relationship between expatriates’ personality traits and their adjustment to international assignments. The International Journal of Human Resource Management.

[CR36] Hunter WC (2021). Cultural representations and experience in tourism: Two forms of mimesis. Journal of Smart Tourism.

[CR37] Hyun MY, O’Keefe RM (2012). Virtual destination image: Testing a telepresence model. Journal of Business Research.

[CR38] Ijsselsteijn, W. A., & Riva, G. (2003). Being there: The experience of presence in mediated environments. In G. Riva, F. Davide, & W. A. Ijsselsteijn (Eds.), *Being there: Concepts, effects and measurement of user presence in synthetic environments* (pp. 4–16). Ios Press

[CR39] Jung, T., Dieck, M. C., Lee, H., & Chung, N. (2016). Effects of Virtual Reality and Augmented Reality on visitor experiences in museums. In *Information and Communication Technologies in Tourism 2016* (pp.621–632)

[CR40] Kang M, Gretzel U (2012). Effects of podcast tours on tourist experiences in a national park. Tourism Management.

[CR41] Kim HC, Hyun MY (2016). Predicting the use of smartphone-based Augmented Reality (AR): Does telepresence really help?. Computers in Human Behavior.

[CR42] Kim MJ, Lee CK, Preis MW (2020). The impact of innovation and gratification on authentic experience, subjective well-being, and behavioral intention in tourism virtual reality: The moderating role of technology readiness. Telematics and Informatics.

[CR43] Kolar T, Zabkar V (2010). A consumer-based model of authenticity: An oxymoron or the foundation of cultural heritage marketing?. Tourism Management.

[CR44] Lee SJ (2017). A review of audio guides in the era of smart tourism. Information Systems Frontiers.

[CR45] Lee S, Phau I, Hughes M, Li YF, Quintal V (2016). Heritage tourism in Singapore Chinatown: A perceived value approach to authenticity and satisfaction. Journal of Travel & Tourism Marketing.

[CR46] Li X, Shen H, Wen H (2016). A study on tourists perceived authenticity towards experience quality and behavior intention of cultural heritage in Macao. International Journal of Marketing Studies.

[CR47] Lin YC, Liu YC (2018). Deconstructing the internal structure of perceived authenticity for heritage tourism. Journal of Sustainable Tourism.

[CR48] Lindell MK, Whitney DJ (2001). Accounting for common method variance in cross-sectional research designs. Journal of Applied Psychology.

[CR49] Loureiro SMC (2019). Exploring the role of atmospheric cues and authentic pride on perceived authenticity assessment of museum visitors. International Journal of Tourism Research.

[CR50] Malhotra NK, Kim SS, Patil A (2006). Common method variance in IS research: A comparison of alternative approaches and a reanalysis of past research. Management Science.

[CR51] Milman A (2013). Guests’ perception of staged authenticity in a theme park: An example from Disney’s Epcot’s World Showcase. Tourism Review.

[CR52] Moscardo, G. (2009). Understanding tourist experience through mindfulness theory. In M. Kozak, & A. Decrop (Eds.), *Handbook of tourist behaviour: Theory and practice*. Routledge

[CR53] Moscardo G, Pearce PL (1986). Historic theme parks. Annals of Tourism Research.

[CR54] Mura P, Tavakoli R, Pahlevan Sharif S (2017). ‘Authentic but not too much’: Exploring perceptions of authenticity of virtual tourism. Information Technology & Tourism.

[CR55] Nguyen THH, Cheung C (2016). Toward an understanding of tourists’ authentic heritage experiences: Evidence from Hong Kong. Journal of Travel & Tourism Marketing.

[CR56] Nuryanti W (1996). Heritage and postmodern tourism. Annals of Tourism Research.

[CR57] Oghuma AP, Libaque-Saenz CF, Wong SF, Chang Y (2016). An expectation-confirmation model of continuance intention to use mobile instant messaging. Telematics and Informatics.

[CR58] Orru K, Kask S, Nordlund A (2019). Satisfaction with virtual nature tour: The roles of the need for emotional arousal and pro-ecological motivations. Journal of Ecotourism.

[CR59] Park E, Choi BK, Lee TJ (2019). The role and dimensions of authenticity in heritage tourism. Tourism Management.

[CR60] Paszkiewicz A, Salach M, Dymora P, Bolanowski M, Budzik G, Kubiak P (2021). Methodology of implementing virtual reality in education for industry 4.0. Sustainability.

[CR61] Podsakoff PM, MacKenzie SB, Lee JY, Podsakoff NP (2003). Common method biases in behavioral research: A critical review of the literature and recommended remedies. Journal of Applied Psychology.

[CR62] Poria Y, Butler R, Airey D (2004). Links between Tourists, Heritage, and Reasons for Visiting Heritage Sites. Journal of Travel Research.

[CR63] Radermecker ASV (2021). Art and culture in the COVID-19 era: For a consumer-oriented approach. SN Business & Economics.

[CR64] Refsland, S. T., Ojika, T., Defanti, T., Johnson, A., Leigh, J., Loeffler, C., & Tu, X. (1998). Virtual great barrier reef: A theoretical approach towards an evolving, interactive VR environment using a distributed DOME and CAVE System. In J. C. Heudin (Ed.), *Virtual Worlds* (Vol. 1434, pp. 323–336). Springer. 10.1007/3-540-68686-X_31

[CR65] Reisinger Y, Steiner CJ (2006). Reconceptualizing object authenticity. Annals of Tourism Research.

[CR66] Salamone FA (1997). Authenticity in tourism. Annals of Tourism Research.

[CR67] Sanchez-Vives MV, Slater M (2005). From presence to consciousness through virtual reality. Nature Reviews Neuroscience.

[CR68] Schwan S, Dutz S (2020). How do visitors perceive the role of authentic objects in museums?. Curator: The Museum Journal.

[CR69] Sears DO (1986). College sophomores in the laboratory: Influences of a narrow data base on social psychology’s view of human nature. Journal of Personality and Social Psychology.

[CR70] Selwyn, T. (1996). *The tourist image: Myths and myth making in tourism*. Wiley

[CR71] Sharpley, R. (2018). *Tourism, tourists and society* (Fifth edition). Routledge, Taylor & Francis Group

[CR72] Silver I (1993). Marketing authenticity in third world countries. Annals of Tourism Research.

[CR73] Skinner H, Chatzopoulou E, Gorton M (2020). Perceptions of localness and authenticity regarding restaurant choice in tourism settings. Journal of Travel & Tourism Marketing.

[CR74] Slater M, Wilbur S (1997). A framework for immersive virtual environments (FIVE): Speculations on the role of presence in virtual environments. Presence: Teleoperators and Virtual Environments.

[CR75] Sylaiou S, Mania K, Karoulis A, White M (2010). Exploring the relationship between presence and enjoyment in a virtual museum. International Journal of Human-Computer Studies.

[CR76] Trilling, L. (1972). *Sincerity and Authenticity*. Harvard University Press. 10.2307/j.ctvjhzrdp

[CR77] Tussyadiah, I. P., Wang, D., & Jia, C. H. (2016). *Exploring the persuasive power of virtual reality imagery for destination marketing*. Travel & Tourism Research Association: Advancing Tourism Research Globally 25

[CR78] Tussyadiah IP, Wang D, Jung TH, tom Dieck MC (2018). Virtual reality, presence, and attitude change: Empirical evidence from tourism. Tourism Management.

[CR79] Wang N (1999). Rethinking authenticity in tourism experience. Annals of Tourism Research.

[CR80] Waysdorf A, Reijnders S (2018). Immersion, authenticity and the theme park as social space: Experiencing the Wizarding World of Harry Potter. International Journal of Cultural Studies.

[CR81] Wei W, Qi R, Zhang L (2019). Effects of virtual reality on theme park visitors’ experience and behaviors: A presence perspective. Tourism Management.

[CR82] Wu D, Shen C, Wang E, Hou Y, Yang J (2019). Impact of the perceived authenticity of heritage sites on subjective well-being: A study of the mediating role of place attachment and satisfaction. Sustainability.

[CR83] Yi, X., Fu, X., Lin, V. S., & Xiao, H. (2021). Integrating authenticity, well-being, and memorability in heritage tourism: A two-site investigation. *Journal of Travel Research*, 004728752098762. 10.1177/0047287520987624

[CR84] Zhang H, Cho T, Wang H, Ge Q (2018). The influence of cross-cultural awareness and tourist experience on authenticity, tourist satisfaction and acculturation in World Cultural Heritage Sites of Korea. Sustainability.

[CR85] Zhang T, Wen H, Li X (2018). A tourist-based model of authenticity of heritage sporting events: The case of Naadam. Sustainability.

[CR86] Zhou, Q. (Bill), Zhang, J., Zhang, H., & Ma, J. (Eds.). (2015). A structural model of host authenticity. *Annals of Tourism Research*, *55*, 28–45. 10.1016/j.annals.2015.08.003

